# The Final Diagnosis: Evaluating Errors in Cause-of-Death Reporting

**DOI:** 10.7759/cureus.82785

**Published:** 2025-04-22

**Authors:** Karthikeyan Srinivasan, Kumar Dharmendra Singh, Aditi Mehra, Bhumica Dang, Syed R Quadri, Akshi Walecha, Shilpa Ratan, Abid Manzoor, Jaspinder Pratap Singh, Yatin Talwar

**Affiliations:** 1 Medical Records Department, Postgraduate Institute of Medical Education and Research, Chandigarh, IND; 2 Hospital Administration, All India Institute of Medical Sciences, Raebareli, Raebareli, IND; 3 Hospital Administration, Government Medical College Amritsar, Amritsar, IND; 4 Anatomy, Bhagat Phool Singh Government Medical College for Women, Khanpur Kalan, Sonipat, IND; 5 Cardiovascular Anesthesiology, Paras Hospitals, Panchkula, IND; 6 Biochemistry, Shri Mata Vaishno Devi Institute of Medical Excellence, Kakryal, IND; 7 Physiology, Shri Mata Vaishno Devi Institute of Medical Excellence, Kakryal, IND; 8 Forensic Medicine, Shri Mata Vaishno Devi Institute of Medical Excellence, Kakryal, IND; 9 Hospital Administration, Shri Mata Vaishno Devi Institute of Medical Excellence, Kakryal, IND

**Keywords:** cause of death, certification errors, icd guidelines, mortality statistics, public health, retrospective study

## Abstract

Background: Accurate cause of death (COD) certification is essential for public health planning and mortality statistics. Despite legal frameworks and WHO guidelines, documentation errors persist, particularly in North Indian hospitals, affecting data reliability and policy decisions.

Aim: This study aims to assess the accuracy, completeness, and common errors in COD certification, identify factors contributing to documentation deficiencies, and propose measures for improvement.

Methods: A retrospective, cross-sectional study was conducted at Shri Mata Vaishno Devi Institute of Medical Excellence (Medical College & Associated Hospital, SMVDNSH), a tertiary care hospital in North India, which is also a centre of excellence and teaching institute, analysing 1,147 COD certificates issued between March 2023 and March 2024. Certificates were assessed for accuracy based on ICD guidelines, and autopsy reports were reviewed to identify discrepancies. Errors were categorized into major and minor types and analyzed across hospital departments and certifier experience levels. Statistical analysis was performed using IBM SPSS Statistics for Windows, version 17.

Result: Only 119 (10.38%) of the certificates were correctly filled. Major errors were found in 858 (74.80%) certificates, while minor errors were present in 810 (70.66%) certificates. A discrepancy between clinical and autopsy COD was observed in 112 (9.77%) of certificates. During Root cause analysis from the users’ perspective, it was found that out of 125 junior residents, 90 (72%) had made mistakes while filling the certificates, which reduced to 35 (28%) post-training. The highest errors occurred in the emergency (619; 54%), and 321 (28%) certificates were from the ICU.

Conclusion: Significant deficiencies in COD documentation highlight the need for targeted training, stricter adherence to ICD guidelines, and regular audits. Addressing these issues will enhance the accuracy of mortality data and improve public health planning.

## Introduction

Cause of death (COD) refers to the disease or injury responsible for initiating the lethal sequence of events leading to death. A competent COD must be etiologically specific, with the underlying or proximate cause being the fatal condition that directly results in death without interruption by any efficient intervening cause [1]. Immediate causes, including complications and sequelae from the underlying cause, may contribute to mortality but do not absolve the primary cause of its ultimate responsibility. Death mechanisms, which are physiological and biochemical changes resulting from the cause's fatal impact, are not etiologically specific and cannot be used as a substitute for the underlying cause [2,3]. Most natural deaths result from diseases such as congestive heart failure, cardiac arrhythmias, septicemia, hepatic failure, and anoxic encephalopathy. Deaths can be broadly categorized as natural or violent, with the latter further classified as accidents, homicides, suicides, or undetermined fatalities. 

Accurate COD certification is crucial for reliable mortality statistics, yet deficiencies in COD documentation persist globally, including in India [4]. Ensuring quality in death certification is essential for public health planning, policy implementation, and medical research priorities. The World Health Organization (WHO) emphasizes the necessity of accurate birth and death statistics for social and economic planning across various sectors. The International Classification of Diseases (ICD) provides guidelines for classifying unnatural deaths, with specific forms (Form No. 4 for hospital deaths and Form No. 4A for non-institutional deaths) aligning with WHO standards. These forms document immediate and antecedent causes of death while recording other significant but unrelated morbid conditions [4,5]. The RBD Act, 1969, mandates compulsory registration of births and deaths in the country and has been in force since 1st April 1970, with provisions for medical certification by practitioners. The Act outlines the responsibility of medical professionals in issuing COD certificates and prescribes penalties for non-compliance. Legal provisions under the RBD Act define responsibilities for COD documentation. Section 10(2) allows the State Government to require COD certification in prescribed formats, while Section 10(3) mandates medical practitioners to issue COD certificates without fees. Section 17(1)(b) restricts public access to COD details in registration records, ensuring confidentiality. Section 23(3) penalizes non-compliance by medical practitioners with fines, reinforcing accountability in COD certification 6. Despite this legal framework, errors in COD documentation remain prevalent due to insufficient training, lack of access to medical records, and failure to follow ICD guidelines for reporting deaths involving injuries or external causes [6,7]. Addressing these challenges through improved training and stricter hospital processes is crucial for enhancing the accuracy and reliability of COD records.

Despite the legal framework and global guidelines, significant gaps remain in the accurate documentation of COD, particularly in North Indian hospitals. Limited research has been conducted to assess the prevalence and types of errors in COD certification in this region. This study aims to bridge this gap by systematically collecting and analyzing COD data from multiple hospitals across North India. By evaluating the accuracy, completeness, and adherence to ICD guidelines, this research will identify common documentation errors and their underlying causes [8,9]. Given the critical role of COD records in public health and policy-making, this study is essential for improving the reliability of mortality statistics and enhancing medical certification practices in the region.

## Materials and methods

Study design

A retrospective, cross-sectional study was conducted at Shri Mata Vaishno Devi Institute of Medical Excellence (Medical College & Associated Hospital, SMVDNSH), a tertiary care hospital in North India, which is also a centre of excellence and teaching institute, analyzing 1,147 COD certificates issued between March 2023 and March 2024. Ethical clearance was obtained from the Institutional Ethics Committee of SMVDNSH, and permission was granted by the Medical Superintendent to access COD certificates stored in the Medical Records Department.

Data collection

At the time of death, the concerned clinical department issues two copies of the COD certificate, out of which one copy is sent to the Registrar of Births and Deaths, and the second copy is retained in the Medical Records Department.

For this study, only in-hospital deaths (i.e., cases documented using Form 4) with post-mortem autopsies performed in our hospital were included for analysis. The COD certificates were assessed for accuracy based on guidelines from the Physicians’ Manual on Certification of Cause of Death (MCCD). In addition, autopsy reports were reviewed and compared with corresponding clinical COD certificates to identify discrepancies. 

The data collected for this study encompassed a comprehensive range of variables, categorized into two main domains: characteristics of the event and characteristics of the deceased. Under the event characteristics, key details such as the date of occurrence and the date of registration were recorded, along with the place of occurrence and the place of registration. Further granularity was captured through information on the locality of occurrence and whether the event took place in an urban or rural setting. In addition, the cause(s) of death were documented, supported by the certifier’s designation and the type of medical certification involved in the process. On the other hand, the characteristics of the deceased included demographic and personal information such as the date of birth, age at the time of death, gender, and marital status, as well as the place of usual residence. This structured yet detailed approach to data collection ensured a holistic understanding of both the contextual and individual factors associated with each recorded event. The types and descriptions of errors that were collected are tabulated in Table 1.

**Table 1:** Types and description of errors

Error type	Specific errors	Description
Type 1 Errors	Non-agreement between clinical and autopsy-based COD	Non-agreement between clinical and autopsy-based COD
Type 2 Errors (major)	Incomplete COD certificates	Missing essential details in the certificate
	Use of nonspecific/vague terms (e.g., "old age," "natural causes")	Lack of precise medical terminology
	Manner/mode of death instead of underlying cause (e.g., "cardiac arrest," "suicide")	Misclassification of the death mechanism as COD
	Errors in personal details (e.g., incorrect name, sex, age)	Demographic inaccuracies
	Incorrect sequencing of causes	Failure to list the immediate, Intermediate, and Underlying causes correctly
	Competing and unrelated causes mentioned together	Presence of unrelated conditions in the same certificate
	Failure to specify external causes in injury-related deaths	Missing details on accidents, poisoning, trauma, etc.
	Errors based on certifier designation (junior residents, senior residents, consultants)	Variability in reporting accuracy among different levels of experience
Type 2 Errors (minor)	Failure to mention the time interval between onset and death	Missing crucial temporal information
	Missing physician details (e.g., doctor's name, registration number, official seal)	Lack of proper certification credentials
	Use of ambiguous or redundant terms	Language that does not clarify the underlying COD

Analysis of certifier-based variations

A separate pattern was studied by categorizing errors based on certifier designation (junior residents, senior residents, and consultants). This analysis aimed to determine whether the level of clinical experience influences error rates and the quality of COD documentation. Identifying such patterns could highlight the need for targeted training among different levels of medical professionals.

Analysis of hospital-specific variations

Deaths were further categorized based on the specific hospital area in which they occurred, as different units present varying levels of clinical complexity and workload. The study examined COD documentation from the a) emergency department, where critically ill patients or trauma cases are managed; b) operating theatres (OT), for perioperative deaths and surgical complications; c) intensive care unit (ICU), where patients with severe and multi-organ failure are treated; d) general wards, covering medical and surgical inpatients under routine care; e) high-workload wards, including departments with frequent admissions and critical cases, such as medicine and surgery wards; and f) dermatology and psychiatry.

This categorization will help assess whether the workload and clinical setting influence error rates in COD certification, providing insights into systemic issues affecting documentation accuracy.

Data analysis and public health impact

All data were anonymized and entered into SPSS Statistics for Windows, Version 17.0 (released 2008, SPSS Inc., Chicago) for statistical analysis. The frequency and distribution of errors were evaluated, with subgroup analysis performed based on the designation of the certifying physician (junior resident, senior resident, consultant) and the hospital area where the death occurred (emergency, ICU, OT, etc.). The study also assessed the public health impact of COD reporting errors, including distorted mortality trends leading to misclassification of CODs and compromised epidemiological data affecting disease surveillance and health policy formulation. Complete confidentiality was maintained throughout the study, and no interventional methods were used.

## Results

This cross-sectional study analyzed 1,147 death certificates to evaluate the accuracy and completeness of COD documentation. Errors were identified based on predefined criteria, including missing or incorrect information, discrepancies with autopsy reports, and misclassification of causes. The data were further categorized based on the type of errors, the level of training of the certifying doctors, and the hospital departments where errors were most frequent. In addition, the impact of orientation lectures on improving documentation accuracy was assessed. The findings highlight significant deficiencies in COD certification, underscoring the need for structured training programs and regular audits to enhance documentation quality. The distribution of errors in COD documentation is shown in Table 2.

**Table 2:** Distribution of errors in cause-of-death (COD) documentation

Category	Number of certificates	Percentage
Correctly filled certificates	119	10.38%
Certificates with major errors	858	74.80%
Certificates with minor errors	810	70.66%
Different causes of death in the autopsy report	112	9.77%
Missing duration between the onset and death	293	25.55%
Omission of the antecedent cause of death	233	20.32%

Only 119 certificates (10.38%) were correctly filled. Major errors were found in 858 (74.80%) and minor errors in 810 (70.66%). One hundred twelve cases (9.77%) had differing COD in autopsy reports. Meanwhile, 293 certificates (25.55%) lacked duration details, and 233 (20.32%) omitted antecedent causes.

Out of 125 junior residents, 90 (72%) mistakes were observed prior to the training, which reduced to 35 (28%) post-training. Out of 100 senior residents, errors dropped from 21 (21%) to 13 (13%), while consultants (7%) showed no change as they did not receive any training. The findings have been summarized in Table 3.

**Table 3:** Errors in cause of death (COD) documentation based on certifier category

Category	Errors before training	Errors after training
Junior residents (n = 125)	90 (72%)	35 (28%)
Senior residents (n = 100)	21 (21%)	13 (13%)
Consultants (n = 50)	4 (7%)	4 (7% (as no training was given))

When the root cause analysis (RCA) of COD analysis was done for the prevalence in the respective areas of the hospital, it was found that most errors occurred in emergency & trauma (619; 54%), followed by ICU (321; 28%), busy wards (114; 10%), OTs (55; 5%), and other areas (38; 3%), highlighting the need for targeted training in critical units. The same has been tabulated in Table 4. 

**Table 4:** Representation of errors by hospital departments

Hospital srea	Errors
Emergency and trauma department	619 (54%)
Intensive care unit (ICU)	321 (28%)
Busy wards	114 (10%)
Operating theatres	55 (5%)
Other areas	38 (3%)

## Discussion

Accurate COD documentation is crucial for public health planning, resource allocation, and epidemiological research. However, significant gaps persist in COD certification across North Indian hospitals, leading to unreliable mortality data. This study systematically analyzed 1,147 COD certificates from a tertiary care hospital to identify common documentation errors, their causes, and the role of physician experience in determining error rates. By comparing clinical COD certificates with corresponding autopsy reports, the study aimed to assess adherence to the Physicians’ Manual on Certification of Cause of Death (MCCD) guidelines and highlight areas requiring improvement [9].

The findings revealed substantial deficiencies in COD documentation (Table 3, bar diagram 1). Only 119 out of 1,147 certificates (10.38%) were accurately and completely filled, while major errors were found in 858 certificates (74.80%) and minor errors in 810 (70.66%). Discrepancies between the clinical COD and autopsy findings were observed in 112 cases (9.77%), raising concerns about misclassification. The most common errors included missing time intervals between onset and death (293 cases, 25.55%) and failure to mention the antecedent cause (233 cases, 20.32%). A significant issue was the incorrect listing of "cardiac arrest" as the COD in 228 cases (19.88%) without specifying an underlying cause, reflecting a poor understanding of proper documentation. Error distribution was highest among junior residents (72% error rate before training), which reduced to 28% post-training (Table 4, bar diagram 2). Senior residents improved from 21% to 13% post-training, while consultants showed no change (7%) as they did not receive training. Errors were most prevalent in the Emergency & Trauma Department (54%), followed by the ICU (28%) and busy wards (10%) (Table 5, bar diagram 3), suggesting that high workload areas contribute significantly to documentation inaccuracies.

These findings are consistent with prior research highlighting widespread deficiencies in COD certification across India. Sharma et al. (2024) [10] found that 99.2% of COD certificates at a tertiary care institute lacked the time interval between death and the morbid condition, and 64.6% mentioned competing CODs, similar to the missing antecedent cause in our study. Alam et al. (2024) [11] assessed 410 MCCD forms and reported 86% incorrectly listing the mode of death (e.g., “cardiac arrest”) instead of the underlying cause, closely mirroring our findings of 19.05% errors in COD listing. Kumar et al. (2024) [12] conducted a scoping review across multiple Indian states and found that incorrect sequencing of COD ranged from 12% to 64.7%, aligning with our observation of sequencing errors in numerous certificates. In addition, Lakkireddy DR et al. (2025) [13] examined death certification in Maharashtra and found that junior physicians contributed to 75% of errors due to inadequate training, reinforcing our data that junior residents had the highest error rate pre-training (72%). The global trend also reflects similar issues; studies from Ghana (Akapko et al., 2023) [14] and United Kingdom (Myers KA et al., 2022) [15] reported that over 60% of COD certificates contained errors related to sequencing, terminology, or lack of specificity, indicating that documentation challenges are not unique to India.

The root cause of these documentation errors stems from multiple interrelated factors. A major contributor is the lack of structured training programs on COD certification for medical professionals, especially junior residents, who are often responsible for issuing COD certificates under high-pressure conditions. High workload and time constraints in critical care areas such as emergency and ICU departments lead to rushed documentation, increasing the likelihood of errors (Sharma et al., 2024) [10]. The absence of routine audits and feedback mechanisms further perpetuates poor documentation practices. In addition, the lack of familiarity with MCCD guidelines, particularly among junior doctors, results in improper sequencing of COD and the frequent misclassification of mode of death as the underlying cause (Alam et al., 2024) [11]. Another contributing factor is the overreliance on vague and non-specific terminology (e.g., "natural causes" or "old age"), which reduces the utility of mortality statistics for public health planning. Studies by Kumar et al. (2024) [12] and Adeyinka A (2023) [16] have similarly highlighted these systemic deficiencies, emphasizing the need for targeted interventions to improve accuracy.

Addressing these deficiencies requires a multifaceted approach. Implementing structured training sessions for medical professionals, particularly junior residents, can significantly reduce errors. Regular audits and quality control measures should be integrated into hospital policies to ensure continuous monitoring and feedback. Incorporating COD documentation training into the medical curriculum can provide future physicians with the necessary skills for accurate certification. Despite these recommendations, the study has limitations, including its confinement to a single institution and a relatively small sample size, which may limit the generalizability of findings. Nonetheless, the research underscores the urgent need for systemic reforms in COD documentation practices, which can enhance the reliability of mortality data and inform evidence-based health policies in India.

## Conclusions

This study reveals significant deficiencies in COD certification, with high error rates linked to inadequate training and heavy workloads. Targeted training, stricter adherence to ICD guidelines, and regular audits are essential to improving documentation accuracy. Enhancing COD reporting will strengthen mortality data reliability, ultimately benefiting public health and policy planning.
